# Function and Regulation of the Pyruvate Transporter CstA in *Escherichia coli* †

**DOI:** 10.3390/ijms21239068

**Published:** 2020-11-28

**Authors:** Ana Gasperotti, Stephanie Göing, Elena Fajardo-Ruiz, Ignasi Forné, Kirsten Jung

**Affiliations:** 1 Department of Microbiology, Ludwig-Maximilians-Universität München, 82152 Martinsried, Germany; ana.gasperotti@lmu.de (A.G.); stephanie.goeing@lmu.de (S.G.); e.fajardoruiz@gmail.com (E.F.-R.); 2 Protein Analysis Unit, BioMedical Center (BMC), Ludwig-Maximilians-Universität München, 82152 Martinsried, Germany; ignasi.forne@lrz.uni-muenchen.de

**Keywords:** secondary transporter, pyruvate uptake, global regulator Fis, stationary phase, catabolite repression

## Abstract

Pyruvate is a central metabolite that connects many metabolic pathways in living organisms. To meet the cellular pyruvate requirements, the enterobacterium *Escherichia coli* has at least three pyruvate uptake systems—the H^+^/pyruvate symporter BtsT, and two thus far less well-characterized transporters, YhjX and CstA. BtsT and CstA belong to the putative carbon starvation (CstA) family (transporter classification TC# 2.A.114). We have created an *E. coli* mutant that cannot grow on pyruvate as the sole carbon source and used it to characterize CstA as a pyruvate transporter. Transport studies in intact cells confirmed that CstA is a highly specific pyruvate transporter with moderate affinity and is energized by a proton gradient. When cells of a reporter strain were cultured in complex medium, *cstA* expression was maximal only in stationary phase. A DNA affinity-capture assay combined with mass spectrometry and an in-vivo reporter assay identified Fis as a repressor of *cstA* expression, in addition to the known activator cAMP-CRP. The functional characterization and regulation of this second pyruvate uptake system provides valuable information for understanding the complexity of pyruvate sensing and uptake in *E. coli*.

## 1. Introduction

Pyruvate plays a central role in metabolism. It is formed by the degradation of glucose, alanine, and aromatic compounds, and constitutes the branching point that leads (via acetyl-coA) to the tricarboxylic acid cycle and to fatty acid synthesis, to amino acids, such as alanine, and to gluconeogenesis (via oxaloacetate). Under anaerobic conditions, bacteria can switch to fermentation, and reduce organic compounds to maintain the balance of NAD^+^/NADH. Pyruvate is then reduced to lactate, oxidized to formate, or decarboxylated to acetaldehyde. This makes pyruvate an important node for the switch between aerobic and anaerobic metabolism.

Pyruvate also plays essential roles in regulating bacterial survival and virulence, as pyruvate is a prominent nutrient in eukaryotic host environment [1,2,3,4,5,6]. In recent years, it has been shown that bacteria are able to sense external pyruvate and modulate gene expression accordingly [7,8]. Pyruvate sensing and uptake have also been shown to be crucial for the resuscitation of viable but non culturable (VBNC) *E. coli* cells [9].

Since pyruvate is a central metabolite, the intracellular levels of the compound must be tightly regulated [10]. The excretion of pyruvate, during overflow metabolism, is a common feature of many bacterial species when cultivated under conditions of carbon excess, and contributes to metabolic balancing between carbon uptake and consumption [7,11,12,13,14,15,16]. Not surprisingly, excretion and reuptake of pyruvate are tightly regulated at multiple levels. In *E. coli*, at least two systems for pyruvate uptake have been proposed—one inducible and the other constitutively active [17].

It was already shown that *E. coli* possess two related histidine kinase/response regulator systems (HK/RR), BtsS/BtsR and YpdA/YpdB (recently renamed PyrS/PyrR) that control the expression of the inducible pyruvate uptake systems [13,14,18,19]. BtsS/BtsR is a high-affinity pyruvate sensing system, while pyruvate acts as a low-affinity stimulus for the PyrS/PyrR system. Both systems regulate the expression of the pyruvate/H^+^ symporter BtsT [20], as well as YhjX, a putative low-affinity pyruvate transporter belonging to the major facilitator superfamily [14,21].

BtsT is a secondary transporter with 18 predicted transmembrane helices, which belongs to a small family of transporters named after CstA (the “Putative Peptide Transporter Carbon Starvation Family”, transporter classification TC# 2.A.114) [22]. CstA is an inner membrane protein consisting of 701 amino acids, which was originally described as a putative peptide transporter [23]. It is highly conserved across many species and is involved in biofilm formation, motility, and agglutination [24]. BtsT displays high sequence similarity (75.4%) and identity (61.1%) to CstA [20]. Recently, CstA was identified as constitutively expressed pyruvate transporter by transposon mutagenesis in *E. coli* [21]. Nevertheless, its mode of action and substrate specificity remain unknown. Other members of the CstA family were characterized in vivo, e.g., CstA of *Campylobacter jejuni* [24], CstA, and YjiY of *Salmonella enterica* serovar Typhimurium [25], and the corresponding genes are upregulated under carbon starvation. Knockout mutants have a lower growth rate in the presence of peptides as nitrogen source [23,24]. Furthermore, *cstA* and *yjiY* mutants of *S*. Typhimurium are both impaired in the utilization of several dipeptides [25].

*cstA* expression is regulated at the transcriptional level by the cAMP receptor protein (CRP) under carbon starvation, and negatively regulated at the translational level by the carbon storage regulator CsrA [23,26]. The putative CRP binding site is located about 80 nucleotides upstream from the transcriptional start site, which is unusual for an σ^70^-dependent promoter [27], indicating that the binding of another factor might be required for regulation [23]. Thus far, no other transcriptional regulators have been identified.

In this work, we focused on studying pyruvate uptake in *E. coli*. For this purpose, we generated a triple mutant that is unable to take up pyruvate. We used this mutant to demonstrate that CstA is a specific pyruvate transporter with moderate substrate-binding affinity. CstA is capable of restoring growth on pyruvate as sole carbon (C)-source, as well as chemotactic movement towards it. Our analysis of the *cstA* expression pattern revealed indications that at least two regulators are involved. Using a DNA affinity-capture assay in combination with reporter strains we identified Fis as a repressor for *cstA* transcription.

## 2. Results

### 2.1. Construction of a Mutant that Is Unable to Grow on and Take up Pyruvate

Based on previous work, we hypothesized that *E. coli* possess at least three pyruvate uptake systems, namely BtsT (known pyruvate transporter), YhjX, and CstA [14,20,21]. BtsT was the first pyruvate transporter characterized in *E. coli* [20]. Later, CstA was found also to be involved in pyruvate uptake [21]. Even though the function of YhjX remains elusive, it seems to be involved in pyruvate uptake as well [14,21]. In order to study pyruvate uptake by single transporters in *E. coli*, a mutant deficient in uptake of and growth on pyruvate was required, in order to minimize background activities. For this purpose, we generated the strain *E. coli* MG1655 ∆*btsT* ∆*cstA* ∆*yhjX* (designated as “3Δ mutant”).

**Figure 1:**
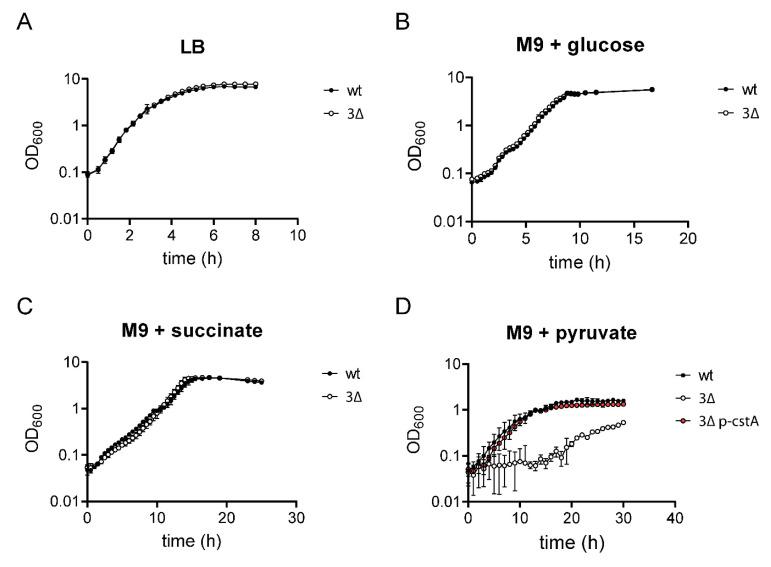
Growth of *E. coli* MG1655 and the triple mutant (3Δ) in media containing different C-sources. Cells of *E. coli* MG1655 (dark circles), the triple mutant (white circles), or the triple mutant complemented with pBAD24-cstA6H (pink circles) were grown in the indicated media at 37 °C under constant agitation. Samples were taken and OD_600_ was measured at different time points. (**A**) LB medium. (**B**) M9 minimal medium with 40 mM glucose. (**C**) M9 minimal medium with 40 mM succinate. (**D**) M9 minimal medium with 40 mM pyruvate. wt: Wild-type strain, 3∆: Triple mutant, 3∆ p-cstA: Triple mutant complemented with pBAD24-cstA6H. The graphs show the means and standard deviations of three independent replicates.

Growth of the 3Δ mutant was tested in media with different C-sources to make sure no other activity was altered. When this mutant was grown in LB medium, it behaved exactly like the wild-type (wt) strain (Figure 1A). The same results were observed when the strains were grown in M9 minimal medium with glucose (Figure 1B) or succinate as sole C-source (Figure 1C). As expected, only when the cells were inoculated into M9 minimal medium with 40 mM pyruvate as sole C- and energy source, the 3Δ mutant was unable to grow (Figure 1D). Single deletion mutants and all possible combinations of double mutants showed no significant growth defect on pyruvate ([20] and Suppl. Figure S1) indicating that each transporter alone can sustain growth on pyruvate.

**Figure 2:**
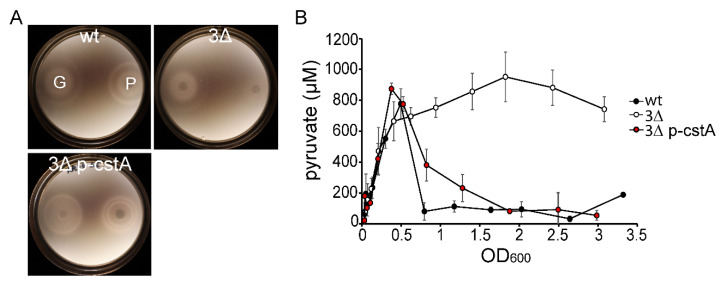
Phenotypic characterization of the 3∆ mutant. (**A**) Chemotaxis. The plug-in-pond assay was utilized to test chemotaxis towards pyruvate and glucose. Agar plugs containing 50 mM pyruvate (P) and glucose (G), respectively, were placed in a petri dish and covered with a suspension of cells in soft agar (0.25%, *w*/*v*). Plates were left to solidify and then incubated at 37 °C for 3 h. The pictures are representative of three independent assays. (**B**) Pyruvate overflow and uptake. *E. coli* MG1655 (wt, black circles), the 3∆ mutant (white circles) and the 3∆ mutant complemented with pBAD24-cstA6H (pink circles) were grown in LB medium at 37 °C, and samples were taken every 20 min. OD_600_ was measured and samples were centrifuged to collect the supernatant. Pyruvate concentrations in the supernatants were determined and values were plotted against OD_600_. Wt: Wild-type strain, 3∆: Triple mutant, 3∆ p-cstA: Triple mutant complemented with pBAD24-cstA6H. The graph shows the means and standard deviations of three independent replicates.

It was previously shown that the influx of sugars influences chemotaxis toward them by modulating the phosphotransferase system (PTS) activity [28]. This applies to other metabolites, including pyruvate, as well. Therefore, we hypothesized that *E. coli* cells need to uptake pyruvate in order to respond chemotactically to it. To test this hypothesis, we assessed the ability of both wt and 3Δ mutant to chemotactically respond to pyruvate. In this case, since the 3Δ mutant is unable to grow on pyruvate, we assessed chemotaxis using the plug-in-pond assay so that the attractant gradient is not generated metabolically. Cells grown in LB were washed with M9 medium lacking a C-source, mixed with warm soft agar, and placed in a petri dish containing two plugs (one containing glucose, and one with pyruvate). After three hours, chemotaxis rings were observed. As shown in Figure 2A, *E. coli* is able to react chemotactically to both glucose and pyruvate, but the 3Δ mutant is impaired in its response to pyruvate (Figure 2A).

In order to demonstrate that the observed defects in growth and chemotaxis are due to the inability of the 3Δ mutant to uptake external pyruvate, we quantified the amounts of extracellular pyruvate present in a culture of the 3Δ mutant growing in LB-medium. For this purpose, we took samples every 20 min and measured the concentration of pyruvate released into the medium due to overflow metabolism (Figure 2B). Pyruvate excretion was not affected by the absence of all three transporters, as indicated by the increase in the external pyruvate concentration in both wt and 3∆ mutant cultures. When overflow ceased, and starvation gradually set in, only the wt strain was able to transport the pyruvate back into the cells, as seen by a decrease in the extracellular concentration (Figure 2B, black circles). The 3Δ mutant, however, was unable to take up pyruvate, and the extracellular concentration of pyruvate remained constant (approximately 800 µM) over time (Figure 2B, white circles).

Taken together, these results indicate that BtsT, CstA, and YhjX are required for pyruvate uptake and necessary for growth on pyruvate as sole C- and energy source.

### 2.2. CstA Restores Growth and Chemotaxis towards Pyruvate

To assess the function of CstA as a pyruvate transporter, we transformed the 3Δ mutant with either pBAD24 (control) or pBAD24-cstA6H (p-cstA) to check whether *cstA* alone can complement the previously described phenotypes. All the experiments were conducted in the absence of inducer (arabinose), as the leakiness of the P_BAD_ promoter should provide a sufficient amount of CstA to allow complementation. Indeed, the 3Δ mutant carrying *cstA in trans* grows at the wild-type rate on M9 minimal medium with pyruvate as sole C- and energy source, indicating the full restoration of pyruvate transport (Figure 1D). Our previous results showed that pyruvate must be taken up in order to function as a chemo-effector. Therefore, we tested the ability of the complemented mutant to respond chemotactically towards pyruvate. When complemented with *cstA*, chemotaxis towards pyruvate was fully restored (Figure 2A). We also measured the extracellular levels of pyruvate produced due to overflow metabolism. The complemented 3Δ mutant and the wt strain were found to show similar decreases in external pyruvate (Figure 2A), indicating that CstA is indeed a pyruvate transporter.

### 2.3. CstA Is a Specific Pyruvate Transporter with Moderate Affinity

**Figure 3:**
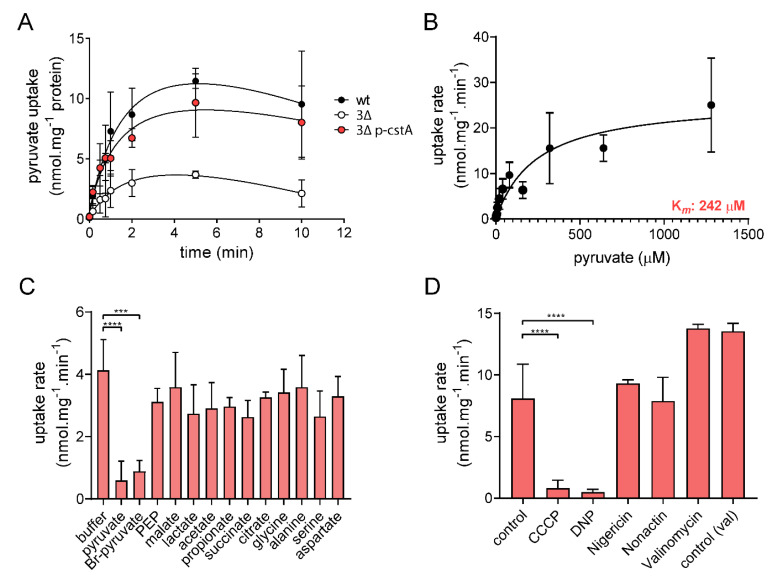
Characterization of pyruvate uptake mediated by CstA in intact cells. (**A**) Time course of pyruvate uptake by *E. coli* strains. Rates of [^14^C]pyruvate uptake were measured at a final pyruvate concentration of 10 µM at 15 °C in *E. coli* MG1655 (black circles), the triple mutant (3∆, white circles), and the triple mutant complemented with pBAD24-cstA6H (3∆ p-cstA, pink circles). (**B**) The K*_m_* value was determined by quantification of the initial rate of pyruvate uptake by CstA in the presence of increasing concentrations of pyruvate. The values were corrected by subtracting the diffusion rates (i.e., uptake rate measured for the 3∆ mutant). The best-fit curve was determined by nonlinear regression using the Michaelis–Menten equation. (**C**) Substrate specificity. The effect of the different substrates on pyruvate uptake was measured by simultaneously adding 1 mM substrate and 10 µM [^14^C]pyruvate. (**D**) Effects of the indicated protonophores and ionophores on pyruvate uptake by CstA. Cells were preincubated at room temperature with the inhibitors for 30 min before adding 10 µM [^14^C]pyruvate. Control: Transport activity in Tris/MES buffer. Control (val): Transport activity in phosphate buffer, used to assess valinomycin effect (see Methods). All experiments were performed in triplicate; the error bars indicate the standard deviations of the mean. One-way ANOVA (multiple comparisons) was performed using GraphPad Prism, comparing each treatment to the control. Significant differences: **** *p* < 0.0001, *** *p* < 0.001.

To characterize CstA biochemically, uptake of [^14^C]pyruvate into intact cells was measured. To this end, the 3Δ mutant was transformed with either pBAD24 or pBAD24-cstA6H (p-cstA) and pyruvate uptake was analyzed by rapid filtration assay. To avoid fast metabolization, all assays were performed at 15 °C. The wt strain showed a linear rate of pyruvate uptake for 60 s before reaching saturation (Figure 3A). Indirect methods demonstrated that the 3Δ mutant is unable to take up pyruvate (Figure 2B), although small amounts of [^14^C]pyruvate were detected in these cells (Figure 3A). This could be explained by simple diffusion of the protonated form of pyruvate or the presence of unspecific transporters that are not able to support growth. Mutant cells complemented with *cstA* displayed similar uptake rates to the wt indicating a full complementation (Figure 3A).

To determine the K*_m_* for pyruvate uptake, we quantified the initial rate of pyruvate uptake by CstA in the presence of different initial concentrations of pyruvate (Figure 3B). To properly calculate the uptake due to CstA, the rates were corrected for the background values measured in the 3Δ mutant transformed with the empty vector. The K*_m_* of CstA for pyruvate in intact cells was estimated to be 242 µM—a rather moderate level of substrate affinity compared to that of BtsT (K*_m_* 16.5 µM) [20].

The specificity of CstA was also evaluated by adding a 100-fold excess of several compounds to test for their ability to compete for the pyruvate binding site (Figure 3C). Only pyruvate itself or Br-pyruvate (a synthetic analogue) were found to act as competitors. None of the other compounds used significantly reduced the rate of pyruvate uptake. Like BtsT [20], CstA seems to have an extremely narrow substrate specificity.

Considering that previous observations revealed that pyruvate transport by BtsT is driven by the proton motive force [20] and that BtsT and CstA share high sequence similarity, we tested the effect of various ionophores on pyruvate uptake by CstA. We used 2,4-dinitrophenol (DNP), carbonyl cyanide m-chlorophenyl hydrazone (CCCP), nonactin, valinomycin, and nigericin for this purpose (Figure 3D). DNP and CCCP are hydrophobic protonophores, valinomycin is a highly selective ionophore for K^+^, while nonactin forms complexes with K^+^, Na^+^, NH^+^_4_, and nigericin promotes potassium-proton antiport. Pyruvate uptake by CstA was only affected by CCCP and DNP (Figure 3D), indicating that transport depends on a proton gradient.

### 2.4. CstA Is Expressed in Late Exponential and Stationary Phase

**Figure 4:**
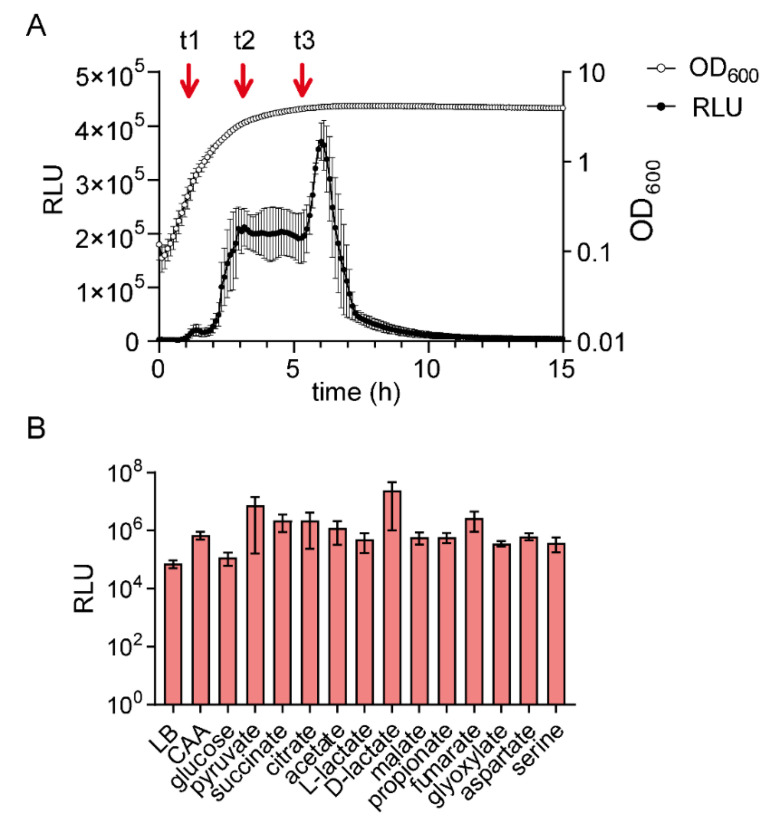
Activation of the *cstA* promoter under various growth conditions. The promoter region of *cstA* (300 bp upstream the gene) was cloned into a reporter plasmid containing the *luxCDABE* operon of *P. luminescens*. *E. coli* MG1655 cells were transformed with this plasmid and grown at 37 °C in the indicated media. Luminescence levels and OD_600_ was measured over time. (**A**) Expression pattern of *cstA*. Luminescence normalized to an optical density (OD_600_) of 1 (RLU) and growth of cells in LB medium over time. The arrows indicate the time points (t1, t2 and t3) at which samples were collected for DNA affinity-capture assay. (**B**) Expression pattern of *cstA* in cells grown in M9 minimal medium supplemented with 40 mM of the indicated C-sources. The maximal luciferase activity normalized to an optical density (OD_600_) of 1 (RLU) served as the measure for *cstA* expression. The histogram shows the maximal levels of *cstA* expression recorded in each case. All experiments were performed in triplicate, and the error bars indicate the standard deviations of the mean. CAA, casamino acids.

The results reported so far were obtained with a 3∆ mutant in which *cstA* was expressed under the control of the P_BAD_ promoter. CstA was recently reported to be a constitutively expressed pyruvate transporter [21]. Indeed, previous results showed that *cstA* expression is under control of σ^70^, the housekeeping sigma factor [29]. However, in a *lacZ* reporter strain, *cstA* expression was also found to be induced under nutrient limitation, and the CRP-binding site was identified in the promoter region of the gene [23]. To analyze the regulation of *cstA* expression in more detail, we generated a reporter strain in which the promoter region of *cstA* (300 bp upstream of the starting codon) was fused to the *luxCDABE* operon of *Photorhabdus luminescens* [30]. Cells were grown in LB medium, and *cstA* expression started in late exponential phase and further increased in stationary phase (Figure 4A). It should be noted that bioluminescence in cells usually decreases dramatically when entering the stationary phase. This phenomenon, known as abrupt decrease in luciferase activity (ADLA) [31,32], is caused by a decrease in the availability of reduced flavin mononucleotide (FMNH_2_) and ATP. Despite these limitations, we found strong stimulation of luciferase activity of cells in the stationary phase, suggesting strong promoter activation. These results are consistent with the previous observation that *cstA* is induced under nutrient limitation [23].

When cells were grown in M9 minimal medium with different C-sources, the fold-change of *cstA* expression was always >1000-fold (compared to the background noise of 100 RLU), indicating full activation of the promoter (Figure 4B and Figure S2). Although *cstA* is regulated by CRP, we observed an activation when cells were grown in the presence of glucose (Figure 4B). However, under this condition, *cstA* expression was strongly delayed and only started in the stationary phase, when the glucose was consumed (Figure S2). Considering the sensitivity of the bioluminescent output to the energy state of the cultures, the maximum levels of *cstA* expression were quite similar for all C-sources tested (Figure 4B), which suggests that no external stimuli are required for the activation of this gene. In contrast, a carbon source-specific expression was found for the BtsS/BtsR-dependent expression of *btsT* [13].

In summary, the high expression level in stationary phase (Figure 4A and Figure S2) indicated the presence of at least a second regulator besides CRP, which was already suggested by Schultz and Martin [23].

### 2.5. Identification of Fis as a Regulator of cstA

The growth-phase-dependent expression pattern of *cstA* prompted us to search for this putative second regulator. For this purpose, we used a DNA affinity-capture assay [33,34] to identify proteins bound to the promoter region of *cstA*. Cells were grown in LB medium and harvested at the three indicated time points (Figure 4A, t1-3, red arrows). Subsequently, putative regulators were captured out of whole cell extracts with beads conjugated with a DNA fragment encompassing the *cstA* promoter region (positions -300 to -1). The same procedure was done with a DNA fragment of 300 bp within the *cstA* coding sequence, which served as control. LC-MS was used to analyze the samples. All proteins that were found to be enriched (2-fold or higher) in the beads conjugated with the *ctsA* promoter DNA compared to the control are listed in Table 1. Regulators and otherwise unknown proteins were studied in more detail (Table 1).

**Figure 5:**
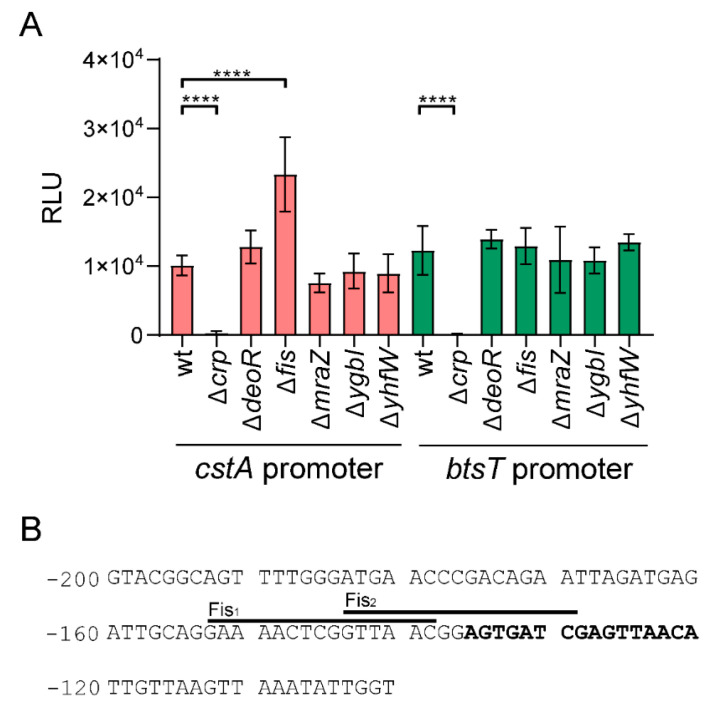
(**A**) Promoter activities of *cstA* and *btsT* in *E. coli* mutants. A luciferase-based reporter assay was used to monitor the promoter activities of *cstA* and *btsT* in the indicated *E. coli* mutants. All strains were transformed with the plasmid pBBR1-*cstA*prom*-lux* or pBBR1-*btsT*prom*-lux*. Bacteria were cultivated in LB medium under aerobic conditions, and the growth and activity of the reporter were continuously monitored. The maximal luciferase activity normalized to an optical density (OD_600_) of 1 (RLU) served as the measure for *cstA* or *btsT* (formerly *yjiY*) promoter activity. All experiments were performed in triplicate, and the error bars indicate the standard deviations of the mean. One-way ANOVA (multiple comparisons) was performed using GraphPad Prism comparing each mutant to the wt, significant differences (**** *p* < 0.0001) were found for Δ*crp* (for both reporter genes) and Δ*fis*. (**B**) Analysis of the promoter region of *cstA*. Fragments of the nucleotide sequence of the *cstA* upstream region (positions −200 to −120) within which the binding motifs for CRP and Fis were identified. The CRP binding site (bold letters) corresponds to the sequence previously published [23]. For Fis, the motif G_-7_N_-6_N_-5_N_-4_R_-3_N_-2_N_-1_N_0_N_1_N_2_Y_3_N_4_N_5_N_6_C_7_, based on Shao et al. [35], was used. Two possible binding sites with the specific characteristics where found (Fis_1_ and Fis_2_), both of which are close to the CRP binding site.

The deletion mutants for the five selected genes (Table 1), and a Δ*crp* mutant were each transformed with the reporter plasmid, and the *ctsA* promoter activity of cells grown in LB medium was measured (Figure S3). As a control, a reporter plasmid carrying the *lux* operon under the control of the promoter region of *btsT* was used [13]. As expected, the absence of CRP completely abolished expression of both transporter genes (Figure 5A). As shown in Figure 5A, of the five regulators identified, only the Δ*fis* mutant showed enhanced luminescence compared to the wt strain, indicating that the Fis protein acts as a repressor for *cstA*. None of the other regulators significantly affected the expression of *btsT* or *ctsA*.

Fis (Factor for Inversion Stimulation) is a DNA-binding protein. In *E. coli*, Fis varies in abundance depending on the growth conditions and growth phase. Fis is most abundant in cells grown in rich medium during early exponential growth, but its level decreases during stationary phase. The role of Fis as a transcriptional regulator has been demonstrated for more than 200 genes [36]. Shao et al. [35] characterized the DNA-binding sequence of Fis in *E. coli* and found a highly variable sequence with four highly conserved positions, G_-7_N_-6_N_-5_N_-4_R_-3_N_-2_N_-1_N_0_N_1_N_2_Y_3_N_4_N_5_N_6_C_7_. Analysis of the promoter region of *cstA* (-300/+0) revealed two binding sites with the previously described characteristics (Figure 5B). This result further corroborates a possible role of Fis in the expression of *cstA*. Interestingly, one of the predicted binding sites for Fis partially overlaps with the binding site of CRP [23] (Figure 5B), suggesting that Fis might also interfere with the binding of CRP in exponentially growing cells.

## 3. Discussion

Pyruvate is a central metabolite under both aerobic and anaerobic growth conditions. During glycolysis, glucose is converted into two molecules pyruvate. Some organisms have the Entner-Doudoroff pathway, in which 2-keto-3-desoxy-6-phosphogluconate is cleaved directly to pyruvate and glyceraldehyde 3-phosphate. The latter molecule is converted to pyruvate by the enzymes of the glycolytic pathway. In addition, bacteria can grow in amino acid-rich media, and alanine, serine, cysteine, glycine, and tryptophan are catabolized to pyruvate. Under these conditions, fast-growing bacteria excrete pyruvate rather than metabolizing it completely [7,11,15]. This so-called overflow metabolism is part of a global physiological response to the protein demands associated with energy production and biomass synthesis [37]. Pyruvate levels seem to reflect the quantitative relationship between carbon and nitrogen availability in the cell, and affect amino acid biosynthesis [16].

Taking uptake and export into account, it is not surprising that both in pro- and eukaryotic organisms the intracellular pyruvate concentration is much lower than the maximum external concentration. It ranges from 40 µM in cells to about 100 µM in plasma and serum, but can reach 2 mM in bacterial supernatants and up to 10 mM in blood of diabetics [7,38,39,40].

Moreover, there is increasing evidence for a role of pyruvate and other α-keto acids in biological fitness and resuscitation of dormant cells in bacterial communities [9,41]. Over the past few years, several pyruvate transporters have been characterized in different microorganisms, such as MctC in *Corynebacterium glutamicum* [42], MctP in *Rhizobium leguminosarum* [43], LrgAB in *S. mutans* [1], PftAB in *Bacillus subtilis* [8] and BtsT in *E. coli* [20].

Two HK/RR systems responsible for pyruvate sensing, BtsS/BtsR and PyrS/PyrR were previously identified [13,14,18,44]. These systems are activated by external pyruvate and induce the expression of two genes each of which codes for a transporter, BtsT and YhjX, respectively. We went on to characterize BtsT as a high affinity pyruvate/H^+^ symporter [20]. Even though the precise function of YhjX remains elusive, there are indications that it might function as another inducible pyruvate transporter [14,21]. Besides these two inducible uptake systems, Hwang et al. [21] showed that the peptide transporter CstA might also be involved in pyruvate uptake.

Here we have created a strain lacking all three of these transporter genes (*E. coli* Δ*btsT* Δ*cstA* Δ*yhjX*), and show that this mutant, unlike any of the single or double mutants, is unable to grow on pyruvate as sole C- and energy source (Figure 1 and Figure S1). These results confirm that each of the three transporters is capable of mediating pyruvate uptake in *E. coli*, although BtsT, CstA, and YhjX probably act with different affinities and specificities. Furthermore, the triple mutant has lost the ability to sense pyruvate as chemoattractant (Figure 2A) and this is most likely due to its inability to take up the compound (Figure 2B). It was previously shown that *E. coli* responds chemotactically to pyruvate via the PTS network, whereby the addition of pyruvate affects protein interactions within the PTS network and these signals are further propagated to the chemotaxis pathway [28]. PTS activity obviously reflects the levels of other metabolites, including glycerol, oxaloacetate, and serine, and the Sourjik group [28] has proposed that sensing of these metabolites might be mediated by the pyruvate to phosphoenolpyruvate (PEP) ratio, which in turn has an impact on the phosphorylation state of the PTS network. Our results suggest that pyruvate uptake also plays a role in this network by regulating the intracellular levels of pyruvate, and therefore modifying the pyruvate/PEP ratio. Further experiments in this direction need to be conducted in order to understand the relationship between pyruvate uptake, PTS network and chemotaxis.

In this study, we have focused on the characterization of CstA as a pyruvate transporter. As mentioned previously, Hwang et al. [21] showed that a mutant lacking *cstA* was less susceptible than the wt strain to the toxic pyruvate analog 3-fluoropyruvate. Our finding that the triple mutant complemented with CstA alone was sufficient to enable this strain to utilize pyruvate as sole C- and energy source and chemoattractant (Figure 1D, Figure 2) is compatible with the previous result. We studied the function of CstA in intact cells and characterized the protein as a specific pyruvate transporter with a moderate affinity for its substrate. The measured K*_m_* value was 242 µM (Figure 3B). The protonophores CCCP and DNP had a significant inhibitory effect on pyruvate transport, indicating that the transport of pyruvate is driven by a protonmotive force. Similar results were described for BtsT, although this transporter has a 15-fold higher affinity for pyruvate than CstA [20]. BtsT and CstA are unusual secondary transporters insofar as they have 18 transmembrane helices. Both belong to the same transporter family (transporter classification TC# 2.A.114) [22] and share high sequence similarity (75.4%) and identity (61.1%). The number of amino-acid substitutions that differentiates them is sufficient to explain the difference between their respective affinities for pyruvate.

Besides their basic function, the two transporters share some regulatory elements. Expression of both genes is regulated by the cyclic AMP receptor protein (CRP), and the carbon storage regulator A (CsrA) post-transcriptionally inhibits synthesis of the transporters [13,23,26]. The main difference in regulation is that *btsT* is tightly controlled by the BtsS/BtsR HK/RR system [13], while *cstA* is under control of the general sigma factor RpoD (σ^70^) [23].

The question of how P*_cstA_* activation is regulated is important for understanding the pyruvate metabolism of *E. coli*. Using a reporter plasmid, we monitored *cstA* promoter activity in cells grown under different conditions. In contrast to *btsT*, expression seems to be independent of the C-source (Figure 4B). Our analysis of the *cstA* expression pattern revealed evidence for the existence of more than one regulator, which had previously been postulated [23]. We were able to identify Fis (factor for inversion stimulation) as a regulator of the expression of *cstA* (Figure 5A). This protein negatively regulates *cstA* as indicated by the fact that *cstA* expression is significantly increased in a ∆*fis* mutant. Moreover, by using a DNA capture assay, we found Fis to be specifically enriched in the promoter region of *cstA* in early-log-phase cells (Table 1, t1). Fis is the most abundant nucleoid-associated protein during the exponential growth phase in rapidly growing cultures [45]. The intracellular Fis levels peak during early exponential growth and then decrease, falling to very low levels in the stationary phase [46]. Analysis of Fis-dependent gene regulation showed that the expression of 231 genes was significantly altered during one or more growth stages, the majority of them being downregulated by Fis [36]. Fis is able to interact specifically with highly variable DNA sequences. The general binding motif G_-7_N_-6_N_-5_N_-4_R_-3_N_-2_N_-1_N_0_N_1_N_2_Y_3_N_4_N_5_N_6_C_7_ has been derived from base substitution analysis, with the −7G, -3R, +3Y, and +7C bases serving as major determinants for high-affinity binding, while the nucleotide combination −4A/+4T severely hinders binding and an AT-rich central region (N_-2_ to N_2_) facilitates Fis–DNA interactions [35]. On examination of the promoter region of *cstA*, we found two possible binding sites for Fis (Figure 5B, Fis_1_ and Fis_2_). Both sites possess the four major determinants for high-affinity binding, but also at least one nucleotide that reduces binding. Therefore, we speculate that Fis has a low to moderate affinity for these two possible binding sites, which could explain why there is only a two-fold increase of *cstA* expression in the Δ*fis* mutant. Both binding sites are in close proximity to the identified CRP binding site (Figure 5B). Therefore, we propose that Fis acts not only as repressor of *ctsA*, but also blocks access of CRP to its binding site in exponentially growing cells. As the cells approach stationary phase, levels of Fis drop, and the regulator is released from the DNA, thereby permitting binding of CRP and activation of *ctsA* expression (Figure 4A, first peak). In stationary phase cells, in which levels of Fis should be negligible, maximum *cstA* expression can be achieved. Our findings suggest that the timing and level of *cstA* activation is dependent on the growth stage of the population.

When *E. coli* cells are grown in LB medium, *btsT* and *yhjX* are expressed in the mid-exponential growth phase [13,14,44], whereas *cstA* is mainly expressed in stationary phase. Therefore, planktonic *E. coli* cells produce at least one pyruvate transporter in all growth stages. In liquid culture, the excretion of pyruvate during overflow metabolism is followed by rapid uptake so that levels of external pyruvate are low in stationary phase (Figure 2B). CstA may be more important in *E. coli* biofilms, which are stratified in exponential- and stationary-phase cells, and allow exchange of pyruvate [47]. The pyruvate uptake system LrgAB in *S. mutans* is also known to be expressed in stationary phase, but is the only system responsible for the uptake of extracellular pyruvate in this species [1].

Functional redundancy of transporters and sensory systems has been shown for several nutrients and may be a usual strategy for many bacteria, allowing them to increase the range of response in constantly fluctuating environments. Growth under suboptimal nutrient concentrations requires adaptations [48,49], and CstA might be part of this adaptation network to scavenge pyruvate. The results presented here add another piece of information to the puzzle of *E. coli’s* tightly and dynamically regulated pyruvate uptake systems.

## 4. Materials and Methods

### 4.1. Bacterial Strains and Plasmids

In this study, we used the strains and plasmids listed in Table 2. The primers used to generate the deletion mutants or plasmids are provided in Table S1.

*E. coli* mutants were generated by using the Quick and easy *E. coli* gene deletion kit, which uses the RED^®^/ET^®^ recombinase system (Gene Bridges). Shortly, an FRT-PGK-gb2-neo-FRT (kanamycin cassette) was amplified by PCR with flanking regions corresponding to each transporter and introduced into the genomic DNA via Red/ET recombination. The kanamycin marker was subsequently removed from the chromosome using a FLP recombinase. For double and triple mutants, the transporter genes were deleted sequentially. For complementation assays and transport studies, the cells were transformed with vector pBAD24-cstA6H, which codes for CstA-6His, and pBAD24 as control. In all cases, no arabinose was added to the culture, and the leakiness of the P_BAD_ promoter allowed sufficient expression of *cstA* for complementation. To test promoter activity, we used plasmid pBBR1-cstAprom-lux, a promoter-based luciferase reporter construct obtained by cloning the promoter region of *cstA* (300 bp upstream the starting codon) in front of the *luxCDABE* operon [30].

### 4.2. Growth Conditions

All strains were grown overnight in LB medium (10 g/L tryptone, 5 g/L yeast extract, 10 g/L NaCl) or M9 minimal medium [53] containing 40 mM of the indicated C-source. When required, media were supplemented with ampicillin (100 µg/mL) or gentamicin (20 µg/mL). Cells from the overnight culture were transferred to the corresponding fresh medium and grown under agitation (200 rpm) at 37 °C. Growth was monitored over time by measuring the optical density at 600 nm (OD_600_).

### 4.3. Determination of the Extracellular Pyruvate Concentration

To determine the pyruvate concentration in the supernatant, *E. coli* cells (MG1655, MG1655 Δ*btsT* Δ*cstA* Δ*yhjX* pBAD24 or MG1655 Δ*btsT* Δ*cstA* Δ*yhjX* pBAD24-cstA) were grown in LB medium with constant agitation at 37 °C. Every 20 min samples were taken and the OD_600_ was determined. The pyruvate extraction procedure was adapted from O’Donell-Tormey et al. [54] with some modifications. Briefly, a 1 mL aliquot was withdrawn from the culture flask and centrifuged at 14,000× *g* for 5 min. Five hundred microliters of the supernatant were transferred to a 2 mL Eppendorf tube containing 125 µL of ice-cold 2 M HClO_4_ and incubated for 5 min on ice. Afterwards, the acid was neutralized with 125 μL of 2.5 M KHCO_3_, and then the precipitated KClO_4_ and proteins were removed by centrifugation at 14,000× *g* for 10 min. For the assay, the supernatant was diluted 1:5 in 100 mM PIPES buffer pH 7.5. The assay was performed as follows: 200 µL of the diluted samples with 200 µM NADH+H^+^ were added into 96-well plates and the absorbance (A1) was measured at 340 nm. Five microliters of 80 U/mL LDH (Roche) were added and then the sample was incubated at 37 °C in the dark for 30 min. The absorbance (A2) at 340 nm was read again. The change of absorbance at 340 nm (ΔA = A1 − A2) was used to calculate the pyruvate concentration. For the standard curve 0, 50, 100, 150, and 200 µM pyruvate in PIPES was used.

### 4.4. Chemotaxis Assay

For the plug-in-pond assay, the three *E. coli* strains (MG1655, MG1655 Δ*btsT* Δ*cstA* Δ*yhjX* pBAD24, or MG1655 Δ*btsT* Δ*cstA* Δ*yhjX* pBAD24-cstA) were grown in LB medium until OD_600_: 0.6–0.8. The plug-in-pond assay was carried according to Reyes-Darias et al. [55] with some modifications. Briefly, bacteria were collected by low-speed centrifugation (800× *g*) and then washed twice with M9 medium (with no C-source) and resuspended at OD_600_: 0.8. A 100 μL aliquot of melted agar (1.5%, *w*/*v* in M9 medium) containing the chemoattractant was placed in a petri dish. After the agar had solidified, 12 mL of bacterial suspension (OD_600_: 0.4 in M9 medium with 0.25% *w*/*v* agar) was poured around the agar plug. Plates were incubated at 37 °C and monitored for up to 3 h.

### 4.5. Promoter Activity Assay

Promoter activity of *cstA* or *btsT* (as control) was explored in vivo with a luciferase-based reporter gene assay. For this purpose, different *E. coli* mutants were transformed with the plasmid pBBR1-*cstA*prom-lux or pBBR *btsT*prom*-lux* [13]. Cells from an overnight culture were inoculated at a starting OD_600_ of 0.05 into LB medium or M9 minimal medium supplemented with 40 mM of different C-sources in 96-well plates. Plates were then incubated under constant agitation at 37 °C. OD_600_ and luminescence were measured in intervals of 10 min for the total period of 18 h (Clariostar). The maximum luminescence levels (relative light units (RLU) expressed in counts per second per OD_600_) were determined for each growth condition.

### 4.6. Transport Measurements with Intact Cells

*E. coli* strain MG1655 Δ*btsT* Δ*cstA* Δ*yhjX* was transformed with pBAD24 or pBAD24-cstA6H. Cells grown in LB medium in the absence of arabinose were harvested in mid-log phase. Cells were washed and resuspended in transport buffer (100 mM Tris/MES (morpholineethanesulfonic acid) pH 7.5, 5 mM MgCl_2_) to an absorbance of 5 (420 nm) thereby adjusting the total protein concentration to 0.35 mg/mL. Uptake of [^14^C]pyruvate (55 mCi/mmol, Biotrend) was measured at a total substrate concentration of 10 µM at 15 °C. At each time point, transport was terminated by the addition of stop buffer (100 mM potassium phosphate pH 6.0, 100 mM LiCl) followed by rapid filtration through membrane filters (MN gf-5 0.4 µm; Macherey-Nagel). Afterwards, the filters were dissolved in 5 mL of scintillation fluid (MP Biomedicals, Santa. Ana, CA, USA), and radioactivity was determined in a liquid scintillation analyzer (PerkinElmer, Downers Grove, IL, USA). To test substrate specificity, [^14^C]pyruvate (10 µM) uptake was tested in the presence of an excess of the corresponding non-radioactive compounds (1 mM). The effects of protonophores and ionophores were tested after preincubation of cells in transport buffer supplemented with 2 mM DNP, 20 µM CCCP, 6 µM nigericin, 10 µM nonactin, or dimethyl sulfoxide (DMSO) (as a control) at 25 °C for 30 min. In the case of valinomycin, cells were preincubated in 100 mM potassium phosphate buffer, pH 7.5, at 25 °C for 30 min, as control [^14^C]pyruvate uptake was measured in the same buffer without valinomycin.

### 4.7. DNA Affinity Capture Assay

To identify putative transcriptional regulators of *cstA*, we used a DNA capture assay [33,34]. A biotinylated P*_cstA_* fragment was generated by PCR using primers labeled with biotin at the 5′ end. As a control, a biotinylated DNA fragment located within the *cstA* coding sequence was used; 600 pmol of the DNA fragments was immobilized with streptavidin-coated magnetic beads (NEB) according to the manufacturer’s instructions. For the preparation of protein extract, *E. coli* MG1655 was cultivated in 800 mL of LB medium until the indicated growth phase. Cells were harvested at 4 °C, washed with cold protein binding buffer B (20 mM Tris pH 8.0, 1mM EDTA, 0.05% (*v*/*v*) TritonX100, 10% (*v*/*v*) glycerol, 1 mM DTT, 100 mM NaCl), resuspended in 8 mL of the same buffer, and disrupted with a French press. The supernatant was incubated with the previously coated magnetic beads at room temperature for 30 min. After extensive washing to remove unspecific bound proteins, the magnetic beads were subjected to trypsin digestion using the iST 8x kit (PreOmics) following the protocol provided by the manufacturer.

### 4.8. Mass Spectrometry

For LC-MS purposes, desalted peptides were injected in an Ultimate 3000 RSLCnano system (Thermo Scientific, Waltham, MA, USA), separated in a 15-cm analytical column (75 μm ID with ReproSil-Pur C18-AQ 2.4 μm from Dr. Maisch) with a 50-min gradient from 5 to 60% acetonitrile in 0.1% formic acid. The effluent from the HPLC was directly electrosprayed into a Qexactive HF (Thermo) operated in data-dependent mode to automatically switch between full-scan MS and MS/MS acquisition. Survey full-scan MS spectra (from *m/z* 375–1600) were acquired with a resolution of R = 60,000 at *m/z* 400 (AGC target of 3 × 10^6^). The 10 most intense peptide ions with charge states between 2 and 5 were sequentially isolated to a target value of 1 × 10^5^ and fragmented at 27% normalized collision energy. Typical mass spectrometric conditions were spray voltage, 1.5 kV; no sheath and auxiliary gas flow; heated capillary temperature, 250 °C; ion selection threshold, 33,000 counts.

MaxQuant 1.6.10.43 was used to identify proteins and quantify them by iBAQ with the following parameters: Database, uniprot_AUP000000625_Ecoli_20200512; MS tol, 10 ppm; MS/MS tol, 20 ppm Da; Peptide FDR, 0.1; Protein FDR, 0.01 Min. peptide Length, 7; Variable modifications, Oxidation (M); Fixed modifications, Carbamidomethyl (C); Peptides for protein quantitation, razor and unique; Min. peptides, 1; Min. ratio count, 2. Identified proteins were considered as interaction partners of the promoter region of cstA if their MaxQuant iBAQ values displayed a log fold change of 2 or higher compared to the control. The mass spectrometry proteomics data have been deposited into the ProteomeXchange Consortium via the PRIDE [56] partner repository with the dataset identifier PXD021798.

## Figures and Tables

**Table 1:** Identification of proteins bound to the *cstA* promoter. List of proteins enriched in the *cstA* promoter compared to the control fragment for each timepoint (t1, t2, t3). The uncharacterized proteins or regulators that were further studied aremarked with *.

Time Point	Protein	UniProt Description	Fold Change
t1	AtpE	ATP synthase subunit c	2.47
	DeoR *	Regulator	1.97
	Fis *	Regulator	3.53
	HemY	Heme metabolic process	1.82
	MraZ *	Regulator	1.85
	RpmG	50S ribosomal protein	3.19
	XseB	Exodeoxyribonuclease 7 small subunit	2.10
	YdjA	Putative NAD(P)H nitroreductase	1.98
t2	AceA	Isocitrate lyase	2.06
	DeoR *	Regulator	2.19
	IhfA	Integration host factor	2.22
	Lpp	Major outer membrane lipoprotein	2.00
	Rph	Truncated inactive ribonuclease PH	1.77
	RplW	30S ribosomal protein S5	1.94
	RpmA	50S ribosomal protein L27	2.85
	RpsT	30S ribosomal protein S20	1.95
	YgbI *	Uncharacterized HTH-type transcriptional regulator	2.08
	YhfW *	Uncharacterized protein	2.70
t3	DeoR *	Regulator	2.00
	JayE	Putative protein from lambdoid prophage	3.34
	Lpp	Major outer membrane lipoprotein	2.01
	RhlB	ATP-dependent RNA helicase RhlB	2.13
	RnpA	Ribonuclease P protein component	2.68
	YcaC	Probable hydrolase YcaC	1.73
	YgbI *	Uncharacterized HTH-type transcriptional regulator	1.83

**Table 2:** List of strains and plasmids.

Strains		
	*E. coli* MG1655	[50]
	*E. coli* BW25113	[51]
	JW5702 (*E. coli* BW25113 Δ*crp*)	[51]
	JW0824 (*E. coli* BW25113 Δ*deoR*)	[51]
	JW3229 (*E. coli* BW25113 Δ*fis*)	[51]
	JW0079 (*E. coli* BW25113 Δ*mraZ*)	[51]
	JW2705 (*E. coli* BW25113 Δ*ygbI*)	[51]
	JW3343 (*E. coli* BW25113 Δ*yhfW*)	[51]
	*E. coli* MG1655 Δ*btsT* Δ*yhjX* Δ*cstA* (3∆)	This study
	*E. coli* MG1655 Δ*btsT* Δ*cstA*	This study
	*E. coli* MG1655 Δ*btsT* Δ*yhjX*	This study
	*E. coli* MG1655 Δ*cstA* Δ*yhjX*	This study
	*E. coli* MG1655 Δ*btsT*	[20]
	*E. coli* MG1655 Δ*cstA*	This study
	*E. coli* MG1655 Δ*yhjX*	[14]
**Plasmids**		
	pBAD24	[52]
	pBAD24-*cstA6His* (pBAD24-cstA6H)	This study
	pBBR1-*cstA*prom-lux	This study
	pBBR *yjiY-lux* (pBBR1-*btsT*prom-lux)	[13]

## Supplementary Materials

Supplementary materials can be found at https://www.mdpi.com/1422-0067/21/23/9068/s1.
