# Supplementary material for: Function and Regulation of the Pyruvate Transporter CstA in Escherichia coli
Source: Int J Mol Sci. 2020 Nov 28;21(23):9068. doi: 10.3390/ijms21239068 (PMC7730263; doi:10.3390/ijms21239068)
Supplement: Supplementary file 1 [file ijms-21-09068-s001.pdf]

## Supplementary Materials

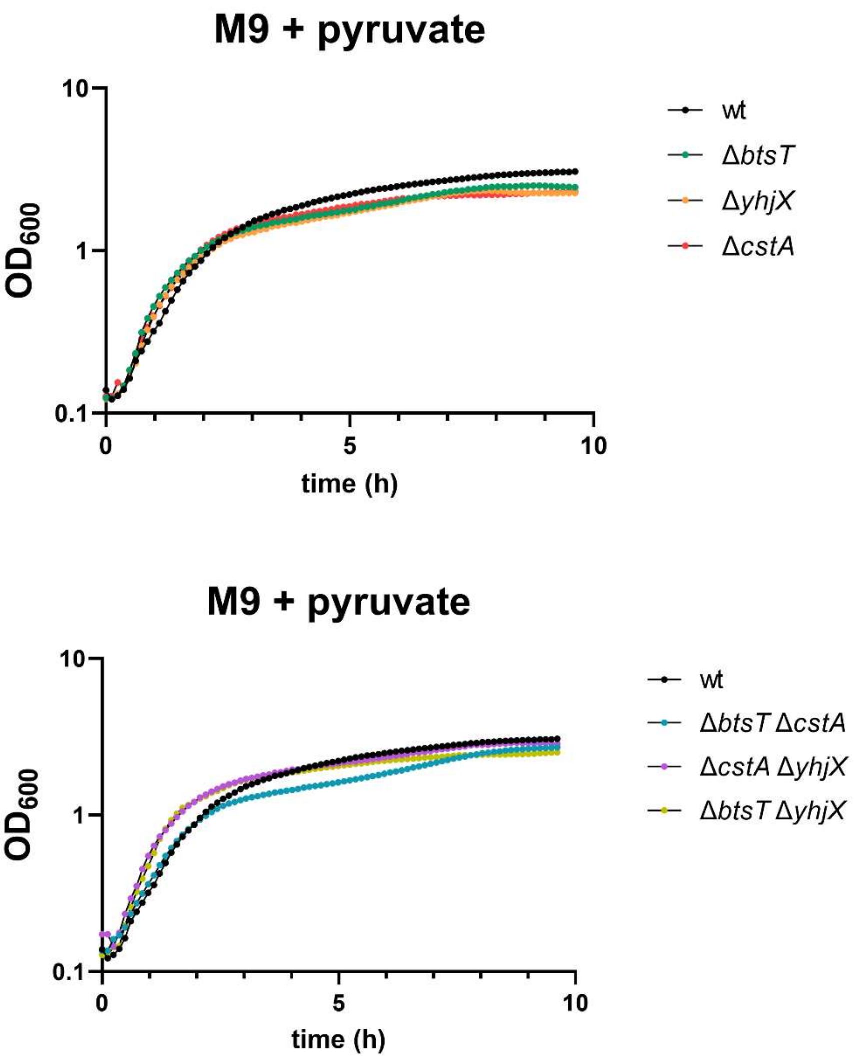


**Figure S1. Growth of *E. coli* MG1655 and the different single and double mutants with pyruvate as C-source**. Cells of *E. coli* MG1655 and the indicated single (upper panel) or double mutants (lower panel) were grown in M9 minimal medium with 40 mM pyruvate as C-source at 37°C under constant agitation. Samples were taken and $OD_{600}$ was measured at different time points. The graphs show the mean of three independent replicates. The standard deviations from the mean were less than 10%.

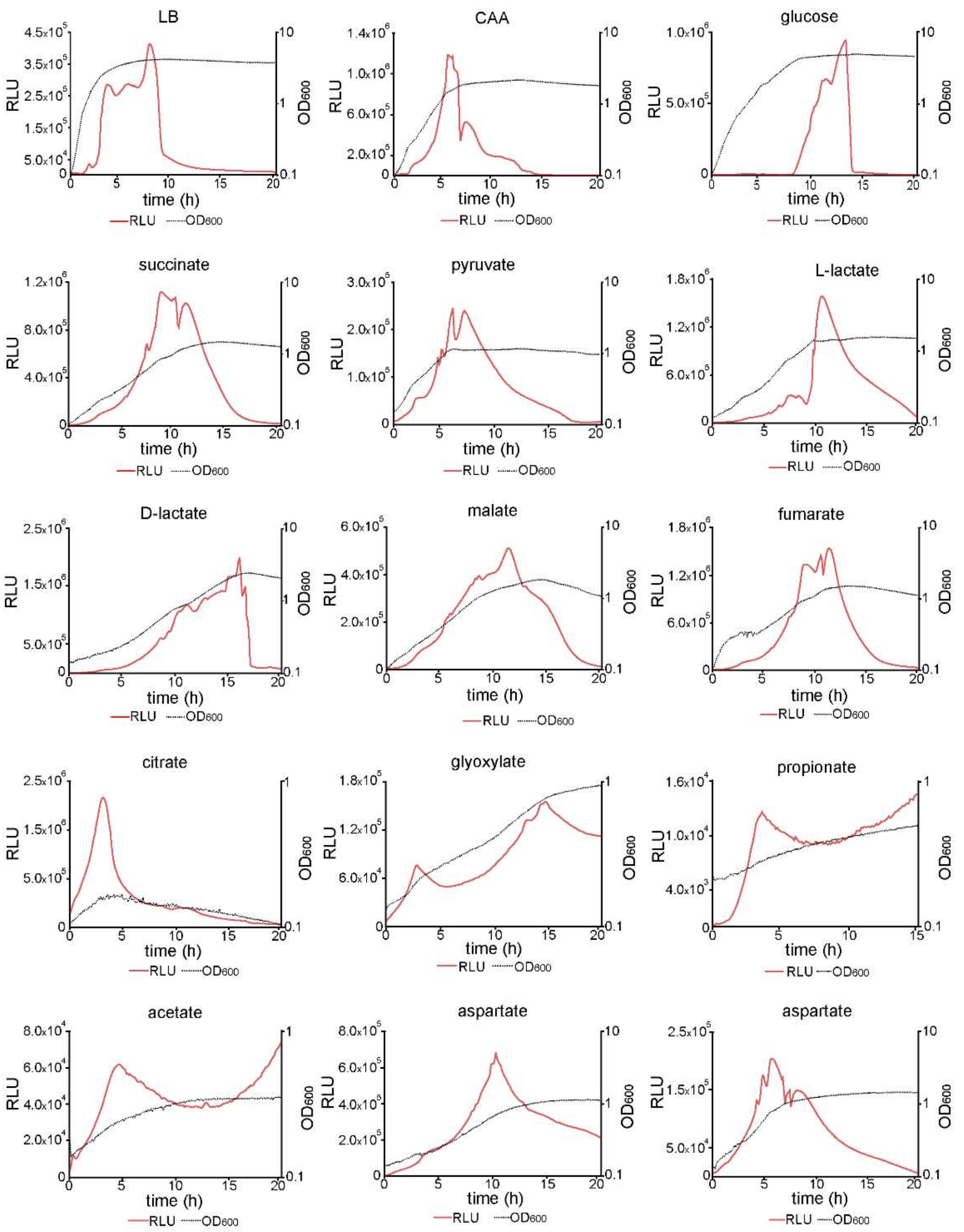


**Figure S2. Activation of the *cstA* promoter under various growth conditions**. *E. coli* MG1655 cells were transformed with pBBR1-*cstA*prom-lux and grown at 37°C in M9 minimal medium supplemented with 40 mM of the indicated C-source. Luminescence levels and $OD_{600}$ were measured over time. Luminescence normalized to an optical density ($OD_{600}$) of 1 (RLU) and growth of cells is plotted over time. CAA, casamino acids.

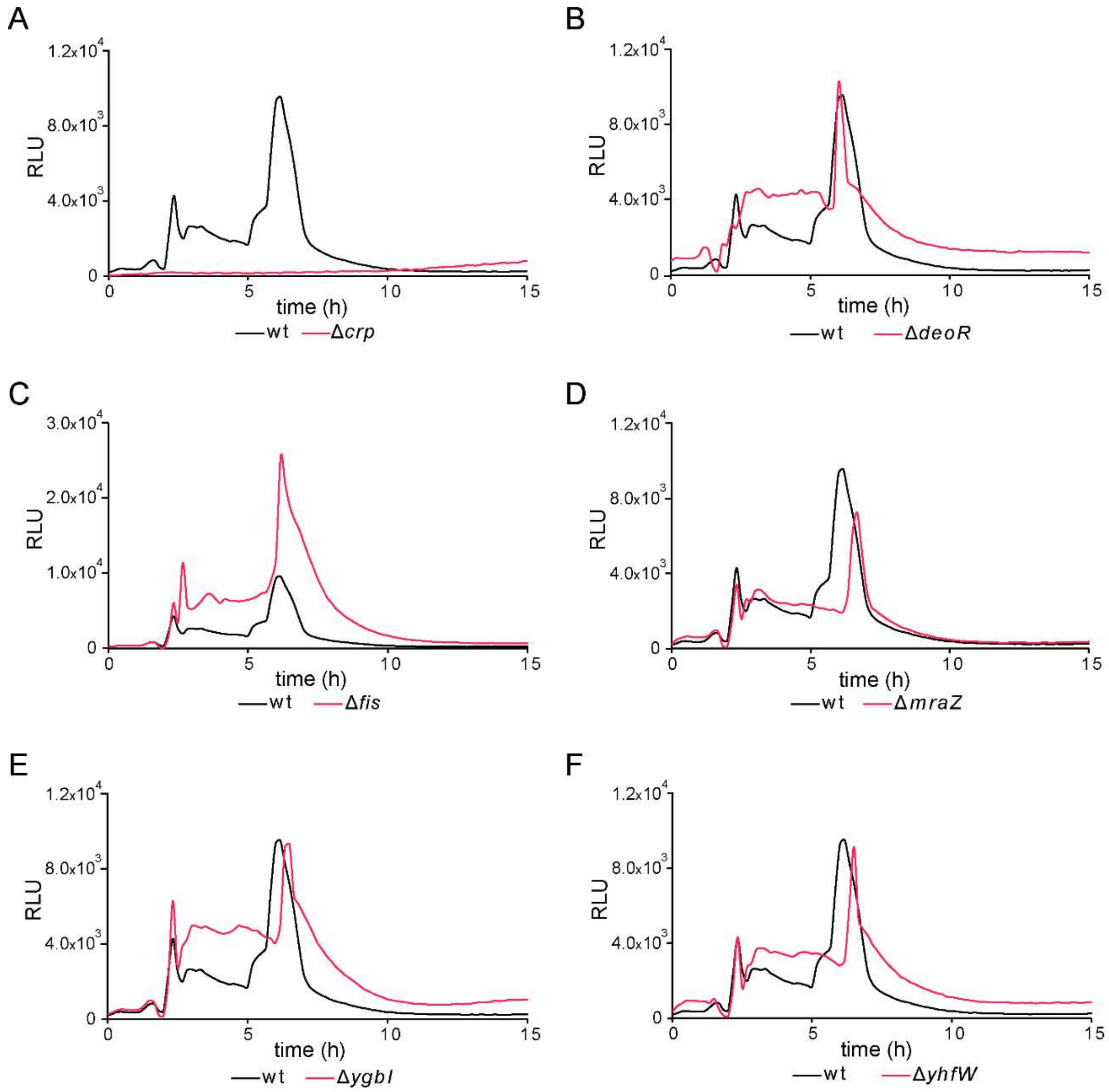


**Figure S3. Promoter activity of *cstA* in different *E. coli* mutants.** A luciferase-based reporter assay was used to monitor the promoter activity of *cstA* in the indicated *E. coli* BW25113 mutants. All strains were transformed with the plasmid pBBR1-*cstA*prom-lux. Bacteria were cultivated in LB medium under aerobic conditions, and the growth and activity of the reporter were continuously monitored. Luciferase activity normalized to an optical density ($OD_{600}$) of 1 (RLU) is plotted over time. A) expression in the Δ*crp* mutant compared to the wt strain. B) expression in the Δ*deoR* mutant compared to the wt strain. C) expression in the Δ*fis* mutant compared to the wt strain. D) expression in the Δ*mraZ* mutant compared to the wt strain. E) expression in the Δ*ygbI* mutant compared to the wt strain. F) expression in the Δ*yhfW* mutant compared to the wt strain.

**Table S1.** List of oligonucleotides used in this work.

| Name | Sequence (5′ – 3′) | Description |
|---|---|---|
| **dYhjX forward** | TTTATTACTGCAGGAATACTG CCATGACACCTTCAAATTATC AGCGTACCAATTAACCCTCAC TAAAGGGCG | Upstream primer for in-frame deletion of *yhjX*, using the Quick and Easy *E. coli* gene deletion kit (Gene Bridges) |
| **dYhjX reverse** | CAGTAGCTCGCGGCTGAGCAT TAAAGGGAGCCATGCGCCTCA CGCAACATTAATACGACTCAC TATAGGGCTC | downstream primer for in-frame deletion of *yhjX*, using the Quick and Easy *E. coli* gene deletion kit (Gene Bridges) |

| | | |
|---|---|---|
| **dbtsT forward** | GGCCAACTATTAATCAATACATGCCAGGTTTTACTATGGATACTAAAAAGAATTAACCCTCACTAAAGGGCG | Upstream primer for in-frame deletion of *btsT*, using the Quick and Easy *E. coli* gene deletion kit (Gene Bridges) |
| **dbtsT reverse** | AGAACAAAGCCCCGCCGAAGCGGGGCTAAACACGGTTAGTGGTGCGAAGATAATACGACTCACTATAGGGCTC | downstream primer for in-frame deletion of *btsT*, using the Quick and Easy *E. coli* gene deletion kit (Gene Bridges) |
| **dcstA forward** | TAACATCTCTATGGACACGCACACGGATAACAACTatgAACAAATCAGGGAATTAACCCTCACTAAAGGGCG | Upstream primer for in-frame deletion of *cstA*, using the Quick and Easy *E. coli* gene deletion kit (Gene Bridges) |
| **dcstA reverse** | CCAACATTCGCCAACATCCCCCCCTCACTCTGACTTTAGTGTGCGCCTTTTAATACGACTCACTATAGGGCTC | downstream primer for in-frame deletion of *cstA*, using the Quick and Easy *E. coli* gene deletion kit (Gene Bridges) |
| **CstA_pBAD_fw** | GGAATTCACCATGGTACCCATGAACAAATCAGGGAAATAC | Gibson assembly fragment 1 forward primer, overlap region of pBAD24 and beginning of *cstA* |
| **CstA_oI_rev** | CCAGGTCAACTGCACGCCGGTAAAG | Gibson assembly fragment 1 reverse primer, internal primer on *cstA* coding region. |
| **CstA_oI_fw** | CTTTACCGGCGTGCAGTTGACCTGG | Gibson assembly fragment 2 forward primer, internal primer on *cstA* coding region. |
| **CstA_pBAD_rev** | GGTCGACTCTAGAGGATCCCCTTAGTGGTGATGGTGATGATGGTGTGCGCCTTTTGCCTGC | Gibson assembly fragment 2 reverse primer, end sequence of *cstA*, 6 his tag and overlap region of pBAD24 |
| **XbaI-CstAprom-Fw** | CTATTCTCTAGACGCGGCGTCTGCCAGCCGCTGCATC | 300 bp upstream starting codon, for *cstA* promoter cloning in pBBR1-lux using XbaI |
| **XhoI-CstAprom-Rv** | CCCCCCCTCGAGAGTTGTTATCCGTGTGCGTGTCCAT | upstream ATG for *cstA* promoter cloning in pBBR1-lux using XhoI |
| **cstApFw** | [Btn]GTCGTTTTCGATGAACAGGGGC | biotinilated forward primer for DNA affinity-capture, *cstA* promoter region. 300 bp upstream of start codon. |
| **cstApRv** | CTGTCCAGACGAGGTATTTCCC | reverse primer for DNA affinity purification, *cstA* promoter region upstream ATG |
| **cstAcFw** | [Btn]GTGGCCTGCTTTATGATCATGG | biotinilated forward primer for DNA affinity purification. Control fragment: *cstA* gene inner region |
| **cstAcRv** | AGGTCAACTGCACGCCGGTAAA | reverse primer for DNA affinity purification. Control fragment: *cstA* gene inner region |
